# A novel GFP nude rat model to investigate tumor-stroma interactions

**DOI:** 10.1186/s12935-014-0146-0

**Published:** 2014-12-21

**Authors:** Ning Yang, Bin Huang, Oleg Tsinkalovsky, Narve Brekkå, Huaiyang Zhu, Lina Leiss, Per Øyvind Enger, Xingang Li, Jian Wang

**Affiliations:** Department of Neurosurgery, Qilu Hospital of Shandong University, Jinan, China; Department of Biomedicine, University of Bergen, N-5009 Bergen, Norway; Brain Science Research Institute, Shandong University, Jinan, China; Neuro Clinic, Haukeland University Hospital, Bergen, Norway; Department of Neurosurgery, Haukeland University Hospital, Bergen, Norway

**Keywords:** Xenograft, Tumor-stroma interaction, Glioblastoma, Breast cancer, Tumor biology

## Abstract

**Backgroud:**

A key strategy for the study of the tumor microenvironment is to implant human tumor cells in an immunodeficient rodent strain ubiquitously expressing a fluorescent marker. Here, a novel nude rat expressing a green fluorescent protein (GFP) transgene was established and engrafted with primary human tumor tissue in order to separate tumor from stromal cell populations for subsequent molecular analysis.

**Methods:**

SD-TG (GFP) 2BalRrrc transgenic rats were crossed with HsdHan™: rnu/rnu Rowett nude rats to develop a GFP expressing immunocompromised rat. PCR and flow cytometry were used to follow the GFP genotype and phenotype in newborns. After three to four generations, animals were implanted with 4 T1 dsRed murine breast cancer cells or primary human glioblastoma (GBM) biopsies to generate xenografts for subsequent separation by fluorescence-activated cell sorting (FACS).

**Results:**

Fluorecence microscopy and reverse transcription-PCR demonstrated that GFP, under the control of the human ubiquitin C promoter, was stably maintained and expressed in diverse organs over several generations. Immunophenotyping of blood samples by flow cytometry confirmed that the immunodeficient features of the parental rat strain, HsdHan™: rnu/rnu, were retained in the GFP nude rat. Both the murine cell line and human GBM biopsies engrafted, and stromal cell populations were isolated from dissociated xenografts by FACS to > 95% purity.

**Conclusions:**

A GFP transgene was stably introduced into a nude rat background in which human and murine cancer cells successfully engrafted. This animal strain provides a novel *in vivo* system for detailed cellular and molecular characterization of tumor-stroma interactions.

## Background

The development of a human tumor is a collaboration between a mutated cell and its microenvironment. The idea that tumor growth was in part dependent on normal cell types originated over 100 years ago when Stephen Paget proposed the “seed and soil” hypothesis in 1889 [1]. Although his original studies addressed the pattern of development of metastasis in human patients, modern studies have revealed that a complex crosstalk between stromal compartments and tumor cells exists at any point during neoplastic progression [2-5].

Stroma itself evolves during cancer development. It is composed of many different cell types, including cancer-associated fibroblasts (CAFs), endothelial cells, and diverse immune cells, as well as extracellular matrix [5]. Tumor cell signalling, survival, proliferation, and even response to chemotherapy are all affected by stroma, but its complexity makes it difficult to attribute specific functional properties to any individual cell type. Therefore, the ability to separate stromal from tumor cells to high purity is essential in order to study tumor-stroma interactions.

One ingenious way to isolate stroma has been to implant tumor cells into an immunodeficient rodent engineered to ubiquitously express a fluorescent marker protein. In the last decades, several green, red, or cyan fluorescent immunodeficient mice models, including two from our group [6,7], have been established where human cancer cells or biopsies successfully engrafted [8-12]. Although these models have been elegantly used to investigate the impact of stromal cell types on multiple aspects of tumor progression, the major disadvantage of xenograft models in mice is tumor size. A substantial amount of material might be required to perform, for example, both genomic and proteomic analyses. The starting cell number is especially important when xenografts are first dissociated into single cell suspensions and subsequently sorted into different cell populations by fluorescence-activated cell sorting (FACS) [6,7]. In addition, any further division of a xenograft based on the complexity of cell types present may require an even greater number of total cells at the start.

CAFs have been recognized as having an important role during the development of epithelial tumor types [13-15], and fluorescent immunodeficient mice have been an important tool in the characterization of the interaction between CAFs and tumor cells. Stroma however, has been not well described for human gliomas. Intracranial xenografts that develop from primary human glioblastomas (GBM) in nude rats recapitulate major characteristics of the disease, including angiogenesis and tumor invasion. In some cases, the xenografts express a stable invasive phenotype independent of angiogenesis over many generations in animals, and therefore, present a unique opportunity to examine molecular differences in histologically distinct stroma [16]. One of our major goals is to characterize these differences in terms of cell type and molecular profiles to aid in our understanding of the development of these tumor types. Transgenic Green fluorescent protein (GFP) rats, which are also available [17-21], are not immunodeficient and thus, are limited in their ability to generate xenograft tumors. As clinically relevant glioma models do develop in immunodeficient nude rats, in order to more effectively study tumor stroma in this model system, we wanted to develop a transgenic nude rat strain ubiquitously expressing GFP. Human GBM biopsies as well as a murine epitheial cancer cell line were implanted in GFP nude rats to generate xenograft tumors. Highly enriched stromal cell populations were isolated from the different tumor types based on fluorescence.

## Results

### Generation of a GFP-expressing nude rat

**Figure 1:**
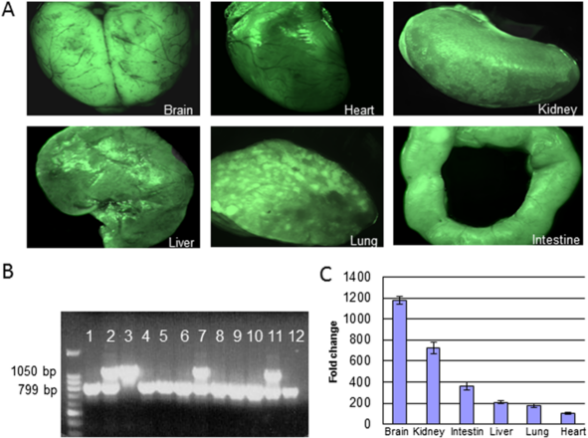
**GFP genotyping and assessment of fluorescence expression in GFP nude rats. (A)** Organs from GFP positive animals visualized under a fluorescence dissecting microscope. **(B)** Confirmation of the GFP genotype by PCR. Homozygous GFP rats displayed a single band at 1050 bp (lane 3), whereas heterozygous rats exhibited bands at both 1050 and 799 bp (lanes 2, 7, and 11). Only a single band at 799 bp was amplified from non-GFP (wild type) rats. **(C)** GFP expression in different organs as estimated by quantitative RT-PCR and expressed as a relative fold change compared to the organ with the lowest expression (heart). Standard error bars are indicated on the columns.

In order to obtain a nude rat expressing GFP, the SD-TG (GFP) 2BalRrrc transgenic rats were crossbred with HsdHan™: rnu/rnu Rowett nude rats. Newborn rats were genotyped at each generation for the presence of the GFP transgene by PCR of genomic DNA isolated from rat tails (Figure 1B). After each cross, heterozygous GFP rats and parental HsdHan™: rnu/rnu Rowett nude rats were again crossbred to obtain heterozygous GFP nude rats and used for tumor implantation experiments after the third generation.

### GFP nude rats stably express GFP in diverse organs

Evaluation of GFP expression was peformed in two ways. Firstly, fresh internal organs harvested from a heterozygous GFP nude rat were examined grossly under a UV dissecting microscope. Organs including brain, heart, kidney, intestine, lung, and liver were all highly fluorescent indicating robust GFP expression (Figure 1A). Secondly, RT-PCR was used to detect and quantify GFP expression at the mRNA level (Figure 1C). RT-PCR revealed that all organs expressed GFP at the transcriptional level. However, the expression levels differed based on tissue type; GFP was expressed at the highest levels in brain and the lowest in the heart relative to GAPDH.

### GFP nude rats exhibit the same immunophenotyping as nude rats

**Figure 2:**
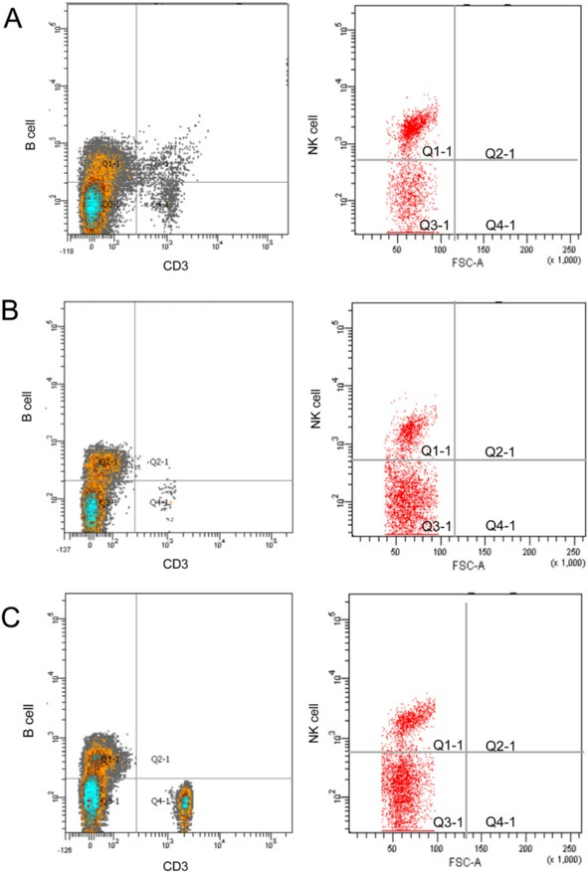
**Immunophenotyping of GFP rat and parental strains by flow cytometry. (A)** GFP nude rat; **(B)** nude rat; and **(C)** immunocompetent rat. Left panels display the distribution of B cells (PE conjugated CD19) and CD3 positive T cells (APC conjugated CD3). Right panels display the distribution of NK cells (Alexa 647 conjugated CD161).

The purpose of the cross was to integrate the GFP transgene into an immunodeficient rat background. To determine whether the homozygous GFP animals retained the immunodeficient features of the parent nude rat strain (HsdHan™: rnu/rnu Rowett nude rat), peripheral blood was collected from GFP nude rats and the two parental rat strains (SD-TG (GFP) 2BalRrrc transgenic and HsdHan™: rnu/rnu Rowett nude rats). White blood cells were isolated, incubated with different fluorescently conjugated antibodies specific for various immune cells types (T, B, and NK cells), and analyzed by flow cytometry. Both the GFP nude rats and HsdHan™:rnu/rnu Rowett nude rats lacked T cells (CD3) but retained B cells and NK cells (CD19 and CD161, respectively; Figure 2). These results indicated that the immune cell profile of the parental immunodeficient animals was preserved in the GFP crossbred nude rats.

### Murine breast and human GBM cell populations engraft in GFP nude rats

One of the most important features for xenograft models is that the host animal does not reject tissue from another species. To determine whether the GFP nude rat provided a favorable environment for engraftment from other species, murine as well as human tumor cells were implanted. Orthotopic implantations were performed at two different locations, in the mammary fat pad and within the brain. For an epithelial tumor type, the murine breast cancer cell line 4 T1 transfected with dsRed was injected into the mammary fat pad. Tumor take was 100% at this site for 4 T1 cells, and tumors had reached 2 cm in size within 21 days.

**Figure 3:**
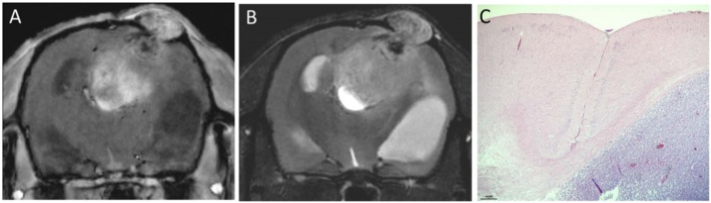
**Engraftment of GBM biopsy in the GFP nude rat. (A)** MRI scan T1 with contrast enhancement (gadolinium contrast reagent). **(B)** MRI scan T2 sequences reveal hydrocephalus of the animal brain. **(C)** H&E staining of the corresponding tumor.

To examine the development of gliomas in the rat brain, spheroids derived from six primary GBMs were transplanted intracranially into GFP nude rats. Animals were monitored by MRI once each week, following implantation, for tumor progression (Figure 3A, B). Tumors developed in rats for 5/6 different GBMs within 6 weeks (Table 1). All animals (n = 13) for these five cases (P2-P6) developed tumors whereas no animal (n = 2) developed a tumor from a single case (P1) within this timeframe. An MRI scan of a typical GBM xenograft revealed a highly aggressive tumor that was invading the contralateral hemisphere of the GFP nude rat. The lateral ventricle was also obstructed, and the tumor was growing out of the skull (Figure 3A, B). H&E staining of the tumor revealed a somewhat circumscribed lesion with local infiltration into the surrounding normal brain tissue (Figure 3C).

**Table 1:** **GBM Tumor take rate in the GFP nude rats**

**Patients**	**Take rate**
P1	0/2 (0%)
P2	3/3 (100%)
P3	2/2 (100%)
P4	2/2 (100%)
P5	3/3 (100%)
P6	3/3 (100%)

### High purity separation of stromal cells from tumors by FACS

**Figure 4:**
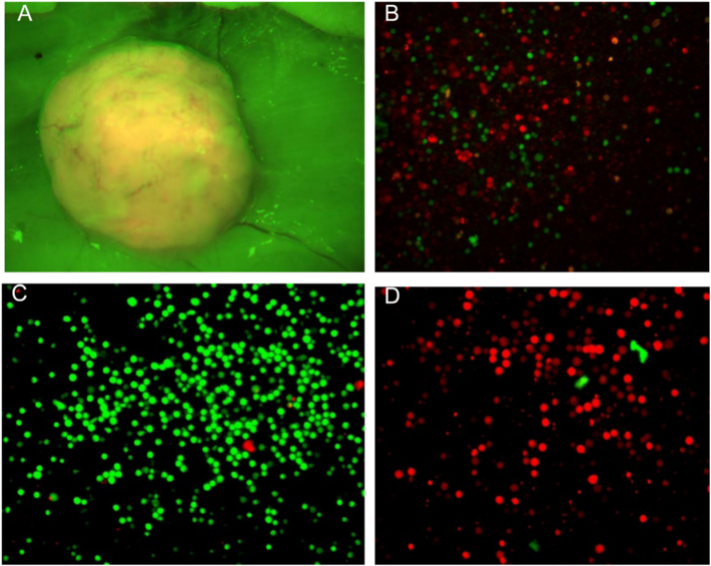
**dsRed 4 T1 xenograft in GFP nude rat. (A)** Subcutaneous 4 T1 dsRed tumor in situ after removing the skin flap of the GFP nude rat (20× magnification). **(B)** Cell suspension of a dissociated 4 T1 tumor displays a mixture of individual GFP expessing host cells with dsRed transfected tumor cells (20× magnification). High purity separation of **(C)** GFP expressing stromal cells (green) and **(D)** dsRed expressing 4 T1 tumor cells (red) after FACS sorting.

The models used allowed separation efficiency to be tested when both tumor and stromal cells were fluorescently labeled or when only stromal cells were labeled. Enzymatic dissociation of dsRed 4 T1 breast cancer xenografts from GFP nude rats (Figure 4A) generated mixed single cell suspensions as demonstrated by the presence of both red (80%-90%) and green fluorescent cells (10%-20%) (Figure 4B). When sorted based on fluorescence, the resultant cell populations displayed high purity (>95%; Figure 4C, D).

**Figure 5:**
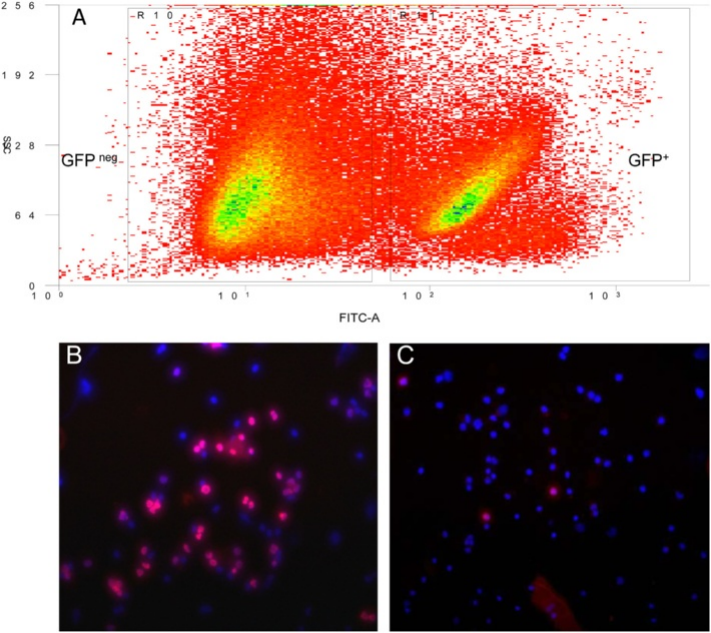
**FACS sorting of cell populations from a GBM xenograft in the GFP nude rat. (A)** Dot plot displays distinction of two cell populations, the GFP negative tumor population (GFP^neg^) and the GFP positive stromal population (GFP^+^), based on X-axis FITC fluorescence distribution. Cell populations were stained with a pan-human specific marker for human nuclear factor (HuNu) before **(B)** and after **(C)** sorting.

To determine whether tumor and stromal cells could be efficiently separated based on a single fluorescent marker, fresh GBM xenografts from the GFP nude rats were enzymatically dissociated into a single cell suspension. Isolated cell populations were obtained based on GFP with high speed FACS (Figure 5A). In order to determine purity, ICC was subsequently performed on sorted cell populations with an antibody specific for the human nucleus. ICC revealed that isolated cell populations were well separated into human (red nuclei) and rat cell types (DAPI; >95%; Figure 5B,C). The sorted cells were quantified, and the total number for cells in each compartment was compared to experiments performed with xenografts from GFP NOD/SCID mice (Table 2). In xenografts from the GFP nude rat, the total number of GFP^+^ sorted stromal cells (> 10^6^) was approximately 10 times greater than in xenografts from GFP NOD/SCID mice. GFP^neg^ human tumor cells comprise 80% of the xenograft in both rats and mice. The total number of GFP^neg^ cells sorted was approximately 5.0×10^6^ and 1.0×10^6^ from xenografts of GFP nude rats and GFP NOD/SCID mice, respectively. These results indicated that increased numbers of cells were routinely sorted from individual xenografts in rats based on fluorescence.

**Table 2:** **Quantification of dissociated/sorted cells from a glioblastoma xenograft in GFP nude rats and GFP NOD/SCID mice**

**Cell compartment**	**GFP nude rats**			**GFP NOD/SCID mice**		
	**R1**	**R2**	**R3**	**M1**	**M2**	**M3**
Total number of cells	1.3×10^8^	1.4×10^8^	1.7×10^8^	1.1×10^7^	1.0×10^7^	1.3×10^7^
GFP^+^ stromal cells	1.0×10^6^	1.3×10^6^	1.5×10^6^	2.0×10^5^	2.0×10^5^	2.5×10^5^
GFP^neg^ tumor cells	5.0×10^6^	5.0×10^6^	5.0×10^6^	1.0×10^6^	1.0×10^6^	1.0×10^6^

## Discussion

Transgene technology allows researchers to introduce a wide range of fluorochrome genes into cells/organisms so that they can be visualized with the desired fluorochrome proteins *in vitro* and *in vivo* and importantly, while they are still living. Immunodeficient animals, such as nude mice, nude rats as well as SCID mice have been extensively used in cancer research because they enable xenografting of human cancer cells or biopsies, and thus generate models that more closely resemble the human tumor phenotype. Together, the two strategies produce powerful experimental tools to characterize the relationship between tumor and microenvironment. In order to begin to define the stroma for human GBM, we established a novel GFP nude rat strain by cross breeding immunocompetent GFP transgenic rats with immunodeficient nude rats. GFP was expressed in all organs examined although mRNA expression levels was dependent on the tissue type. GFP nude rats expressed the immune deficiencies of the parent nude rat, and thus, 4 T1 breast cancer cells as well as human GBM biopies engrafted. Finally, fluorescent stromal cells were separated from non-fluorescent or dsRed cancer cells to high purity by FACS.

In recent years, numerous fluorescent animals, such as mouse, rat, pig, and rabbit, have been established [8,17,22,23]. GFP transgenic rats were first introduced as a model to study organ transplantation in 2001 [17], as well as physiological aspects of a specific cell population or organ [20,24-27]. Athymic nude rats were first reported in the 1970s [28]. Since then, a plethora of experiments exploiting cancer xenografts in nude rats has been reported. The focus of our current experiments is glioma stroma, but fluorescent transgenes have been elegantly used to elaborate the evolution of stroma during tumor progression. Red fluorescent protein (RFP) positive human prostate cancer, colon cancer, breast cancer and fibrosarcoma cells have been implanted orthotopically into GFP nude mice so that tumor-host interactions could be visualized by dual-color imaging [10]. In addition, the origin of some stromal cells from the bone-marrow has been illuminated through experiments where tumor was implanted into non-transgenic SCID mice which received bone marrow transplants from GFP SCID mice [29]. Finally, multiple color proteins have been used to investigate tumor-stroma interactions in real time. Imaging was performed on live animals where cyan fluorescent protein (CFP) expressing nude mice were implanted with dual-colored human pancreatic cancer cells expressing RFP in the cytoplasm and GFP in the nucleus [11].

Even within our group, we have previously established a GFP NOD/SCID mouse strain to investigate tumor-host interactions. Orthotopic implantation of both U87 dsRed GBM and 4 T1 dsRed breast cancer cell lines generated tumors in GFP NOD/SCID mice, and stromal cells were separated by FACS to high purity [6,30]. However, homozygosity of the GFP transgene is a lethal genotype on a NOD/SCID background. Breeding is a cumbersome process as animals must be genotyped at each generation, and only heterozygous pairs used. Ultimately sufficient numbers of GFP NOD/SCID mice are slow to generate. A dsRed NOD/SCID mouse strain was also established as the fluorchrome also has favorable spectral properties at high-emission wavelengths. Homozygosity of the dsRed transgene in NOD/SCID mice is not detrimental to animals as they are completely viable and fertile [7]. For this reason, the dsRed NOD/SCID mice allow for easy breeding and maintenance of the colony.

The GFP nude rat though has several experimental advantages over fluorescent NOD/SCID mice. Firstly, when maintained under specific pathogen-free conditions, the GFP nude rats have a maximum life span of 1.5 to 2 years or more [31] whereas NOD/SCID mice have a life span of 1 year (JAX notes). This lengthy life span of the nude rat provides sufficient time in some instances for studies regarding cancer development of more slow growing tumor types as well as to obtain survival data. In addition, NOD/SCID mice are predisposed to thymic lymphoma as well as wasting and infections which do not occur as frequently in nude rats so that more animals reach experimental endpoints. Secondly, since the immunological characteristics of the GFP nude rat are the same as the nude rat, the absence of T lymphocytes but the retention of B cell linages, the GFP nude rats can be used to study host resistance to experimental infection [31]. Finally, the size of the nude rat facilitates many aspects of *in vivo* experiments simply because more blood and tissue material can be obtained. It is ultimately more feasible to isolate sufficient numbers of diverse cell types for molecular analyses from tumors grown in GFP nude rats. Furthermore, rats are simply much more convenient to use, particularly for intracranial surgeries, such as xenotransplantation of GBM (J. Wang, *personal observation*), and they are less sensitive to overdosing with anesthesia.

## Conclusions

The GFP transgene has been successfully bred onto a nude rat background. Murine and human cancer cells were engrafted in these animals at a high tumor take rate, and the resulting tumors could be separated into stromal and tumor cell compartments to high purity on the basis of fluorescence. Nude rats ubiquitously expressing fluorescent markers are a unique tool for characterization of tumor stroma from diverse human cancers.

## Materials and methods

### Ethics statement

The collection of tumor tissue from patients was approved by the Regional Ethics Committee at Haukeland University Hospital (Project number 013.09; Bergen, Norway). All patients signed informed written consent. This study was carried out in strict accordance with the recommendations in the Guide for the Care and Use of Laboratory Animals of the National Institutes of Health. The protocol was approved by the Committee on the Ethics of Animal Experiments of the University of Bergen (Permit Number: 20135415).

### Cell culture

The murine breast cancer cell line 4 T1 (American Type Culture Collection, ATCC CRL-2539; Rockville, MD, USA) was transfected with a dsRed-expressing lentiviral vector (*Discosoma* sp. red fluorescent protein) [30]. Cells were cultured in RPMI-1640 medium (Bio-Whit-taker; Verviers, Belgium) containing fetal bovine serum (FBS;10%), Penicillin/Streptomycin (100 units/mL), non-essential amino acids (3.2%), plasmocin (0.005 mg/mL; InvitroGen; San Diego, CA, USA), and L-glutamine (400 mol/L; Lonza; Cologne, Germany), and were maintained in a humidified environment at 5% CO_2_ and 37°C.

### Tumor spheroid culture

Six human brain tumor biopsies (GBM) were obtained from the Department of Neurosurgery, Haukeland University Hospital (Bergen, Norway). Spheroids were prepared and selected for intracerebral implantation as previously described [32]. Briefly, surgical samples were minced into 0.5 mm fragments and cultured in agar-coated tissue culture flasks in DMEM containing FBS (10%). Spheroids were maintained in a humidified environment at 5% CO_2_ and 37°C, and the medium was changed once per week. After 2 weeks, ten spheroids with diameters between 200 and 300 μm were selected for intracerebral implantation into rats.

### Animals

SD-TG (GFP) 2BalRrrc transgenic rats (stock no. 0065, Rat Resource & Research Center, University of Missouri; Columbia, MO, USA) harboring the GFP transgene under transcriptional control of the human ubiquitin-C promoter with a woodchuck hepatitis virus posttranscriptional regulatory element (WRE) were crossed with HsdHan™: rnu/rnu Rowett nude rats (stock no. 20196, Harlan Laboratories; Horst, Netherlands) to make first generation GFP heterozygous animals [33]. Inbreeding was subsequently performed between heterozygous GFP rats and the parent HsdHan™: rnu/rnu Rowett nude strain in order to obtain heterozygous GFP nude rats. Experimental animals could be used at the third or fourth generation after confirmation of genotype by PCR.

### DNA extraction and GFP genotyping by PCR

Genomic DNA was extracted from freshly harvested tissue from rat tails according to the manufacturer’s protocols (Extract-N-Amp Tissue PCR Kit; Sigma; St. Louis, MO, USA). A master mix was made which contained Extract-N-Amp PCR Reaction Mix (10 μL), primers 1, 2, and 3 (0.3 μL of each at 25 μM), and sterile water (5.1 μL). Master mix (16 μL) and DNA template (25 ng in 4 μL) were added together for a final reaction volume of 20 μL. Cycling parameters used were the following: 1 cycle 94°C 3 min; 35 cycles 94°C 30 s, 60°C 30 s, and 72°C 75 s; 72°C for 10 min. PCR products were analyzed on a 3% agarose gel in Tris-acetate, EDTA buffer. The wild type fragment migrated at approximately 799 bp, and the transgenic fragment at 1050 bp. Primers used for GFP genotyping of DNA were: R52 int 1 F: 5′-AGCAATGAATAGCCTCTCTCCT-3′, R52 int 1R: 5′-CCCATATGTGCCAAGCACTTTACC-3′, U3R-0: 5′-GTCTGAAGGGATGGTTGTAGCTGT-3′.

### Quantitative Real Time PCR of GFP expression

Diverse tissues from GPF nude and nude rats were harvested and immersed in RNA later solution (Ambion; Austin, TX, USA). Tissue was finely chopped and passed through a 23G needle five times for homogenization. RNA extraction was performed with the RNeasy Plus Mini kit (Qiagen; Hilden, Germany), according to the manufacturer’s protocols. RNA was quantified on the Nanodrop ND-1000 instrument (NanoDrop products; Wilmington, DE, USA). cDNA was synthesized with the iScript Select cDNA synthesis kit (Bio-Rad; Hercules, USA) according to the manufacturer’s protocols. Quantitative real time PCR was performed on cDNA in iQ™ SYBR Green supermix (1×; Bio-Rad, Hercules, CA, USA). cDNAs were amplified in a Bio-Rad iCycler 96 well instrument (Bio-Rad), and reactions for GFP and GAPDH were run in triplicate. Cycling parameters used were: 1 cycle 95°C 5 min; 40 cycles 95°C 30 s, 60°C 40 s, and 72°C 20 s; 1 cycle 72°C 1 min; and 1 cycle 55°C 30 s. The following primers were used to amplify GFP and glyceraldehyde 3-phosphate dehydrogenase (GAPDH): GFP forward primer: 5′-ACGTAAACGGCCACAAGTTC-3′; GFP reverse primer: 5′-AAGTCGTGCTGCTTCATGTG-3′; GAPDH forward primer: 5′-TGTGCAGTGCCAGCCTCGTC-3′, GAPDH reverse primer: 5′-TGCCACTGCAAATGGCAGCC-3′.

### Immunophenotyping

Peripheral blood was collected from the tails of the GFP-nude, and the parent nude and immunocompetent rats. Blood samples were treated with Easylyse buffer to lyse red blood cells (S2364, DAKO; Oslo, Norway), and the remaining cell types were pelleted and resuspended in staining buffer (2% FBS in PBS). Cell suspensions were incubated for 30 min at room temperature with fluorescently conjugated antibodies against rat CD3 (eBioscience; San Diego, CA, USA), CD4, CD8, CD19 (Biolegend; San Diego, CA, USA), and isotype control. Analyses were performed on the FACS Aria II (BD Biosciences; Erembodegem, Belgium).

### *In vivo* experiments

For implantation of breast tumor cells, dsRed-expressing 4 T1 cells were detached from monolayer with trypsin (Lonza, Cologne, Germany) and diluted in PBS. The concentration of 4 T1 cells was adjusted to 1×10^7^ cells/mL. Rats were anesthetized with isofluran (Abbott; Oslo, Norway), and 1×10^6^ cells were injected into the mammary fat pad. Tumor size was measured every two days by caliper. When the diameter of tumors reached 2 cm, rats were sacrificed, and tumors were collected.

Stereotactic intracranial implantations for GBM were performed as previously described [16]. Briefly, rats were anesthetized with a mixture of 10 ml Fentanyl (50 mg/mL; Hameln, Germany) and 1 mL Domitor (1 mg/mL; Orion Pharma, Finland). Rats were fixed in a stereotactic frame (Narishige Group; Tokyo, Japan), a small hole was drilled in the skull, and ten tumor spheroids were slowly injected through a wide bore Hamilton syringe (Hamilton; Reno, NV, USA) into the right frontal cortex. Rats were sacrificed when symptoms developed, and brains were removed. Primary GBM transplants were carefully dissected out from affected brains, and new biopsy spheroids were generated. Spheroids were then seeded on a plastic surface and examined *ex vivo* using a fluorescence microscope (Leica DMI 6000B, Leica Microsystems; Wetzlar, Germany).

### Fluorescence microscopy

Fresh organs were visualized under a fluorescence dissecting microscope (Model C-DSD230; Nikon, Japan) with a UV-filter.

### MRI scanning

MRI scanning was performed on anesthetized animals (1.5% isofluoran mixed with 50% air and 50% O_2_) in a Bruker Pharmascan 7 T MR scanner (Bruker Biospin MRI GmbH; Ettlingen, Germany). An axial T1 weighted MSME sequence (TR 1000 ms, TE 8.7 ms, slice thickness 1 mm, FOV 3.5 cm, matrix size 256 × 256, 20 slices) was acquired before and after administration of subcutaneous injection of contrast agent (1.0 mL of 0.5 mmol/mL Omniscan; Nycomed Amersham, Oslo, Norway). An axial T2 weighted RARE sequence was also acquired (TR 4200 ms, TE 36 ms, slice thickness 1 mm, FOV 3.5 cm, and matrix size 256 × 256, 20 slices).

### Fluorescence-activated cell sorting

4 T1 breast tumors were dissociated into single cell suspensions as previously described [30]. For GBM xenografts, fresh tissue was finely minced and dissociated into a single cell suspension with the Neural Tissue Dissociation kit (Miltenyi Biotec; Bergisch Gladbach, Germany) according to the manufacturer’s protocols. The mixed cell suspensions were subsequently sorted into isolated tumor and stromal cell populations with the FACS Aria II (Becton Dickinson; Erembodegem, Belgium).

### Histological analysis and immunostaining

Rat brains were fixed in 4% formaldehyde immediately following sacrifice. Tissues were embedded in paraffin, and 5 μm sections were prepared and stained with Hematoxylin and eosin (H&E) for histological analysis.

Immunocytochemistry (ICC) was performed on isolated cell populations following FACS. Mouse anti-HuNu (1:100, Millipore, MA, USA) and TXRD-conjugated goat anti-mouse (1:100; Southern Biotech; AL, USA) were used to distinguish human tumor cells from rat host cells. Cells were then mounted in Vectashield mounting medium with DAPI for identification of all nuclei present (Vector Laboratories; Burlingame, CA, USA). Immunocytochemistry was visualized under confocal microscopy (Leica TCS SP5; Leica Microsystems Wetzlar, Germany).

## Abbreviations

GFP: Green fluorescent protein
GBM: Glioblastoma
FACS: Fluorescence-activated cell sorting
CAF: Cancer-associated fibroblasts
H&E: Hematoxylin and eosin
ICC: Immunocytochemistry
RFP: Red fluorescent protein
CFP: Cyan fluorescent protein
